# Diabetes-Associated Fibrin Enrichment in Cerebral Thrombi and Its Procedural Implications for Mechanical Thrombectomy

**DOI:** 10.3390/jcm15208136

**Published:** 2026-10-20

**Authors:** Jang-Hyun Baek, Hyo Suk Nam, JoonNyung Heo, Il Kwon, Byung Moon Kim, Dong Joon Kim, Young Dae Kim

**Affiliations:** 1 Department of Neurology, Kangbuk Samsung Hospital, Sungkyunkwan University School of Medicine, Seoul 03181, Republic of Korea; 2 Department of Neurology, Severance Stroke Center, Severance Hospital, Yonsei University College of Medicine, Seoul 03722, Republic of Korea; 3 Integrative Research Center for Cerebrovascular and Cardiovascular Diseases, Yonsei University College of Medicine, Seoul 03722, Republic of Korea; 4 Division of Interventional Neuroradiology, Department of Radiology, Severance Stroke Center, Severance Hospital, Yonsei University College of Medicine, Seoul 03722, Republic of Korea

**Keywords:** diabetes, fibrin, thrombus composition, mechanical thrombectomy, acute ischemic stroke

## Abstract

**Background/Objectives:** Diabetes is associated with altered fibrin biology, but its relationship with fibrin content in cerebral thrombi and the procedural relevance of this relationship during mechanical thrombectomy remain unclear. We investigated the association between diabetes and fibrin content in thrombectomy-retrieved cerebral thrombi and whether diabetes-associated differences in fibrin content mediated procedural outcomes of mechanical thrombectomy. **Methods:** We retrospectively analyzed patients with acute ischemic stroke who underwent mechanical thrombectomy and had retrieved thrombi available for histological analysis. Red blood cell, platelet, and fibrin contents were quantified immunohistochemically. The association between diabetes and fibrin content was evaluated using multivariable linear regression, with complementary sensitivity analyses based on intravenous tissue-type plasminogen activator administration and atrial fibrillation status. Mediation analyses were performed to estimate the indirect effects of diabetes on procedural outcomes through diabetes-associated changes in fibrin content. **Results:** Among 312 patients, 98 (31.4%) had diabetes. Patients with diabetes had greater fibrin content than those without diabetes (median, 37.0% [interquartile range, 26.4–52.9] vs. 30.9% [17.7–45.0]; *p* = 0.004), whereas red blood cell and platelet contents did not differ significantly between the groups. Diabetes remained associated with greater fibrin content in the primary adjusted analysis (β = +6.68 percentage points; 95% confidence interval [CI], 2.19–11.18; *p* = 0.004), and the association persisted after additional conditioning on initial glucose and in sensitivity analyses. Among patients with diabetes, neither HbA1c nor initial glucose was associated with fibrin content. Mediation analyses did not identify a detectable indirect effect of diabetes through fibrin content on successful reperfusion, complete reperfusion, first-pass effect, number of passes, or the requirement for ≥3 passes. **Conclusions:** Diabetes was independently associated with greater fibrin content in cerebral thrombi retrieved during mechanical thrombectomy, supporting the presence of a diabetes-associated fibrin-enriched thrombus phenotype. However, no detectable indirect effect of diabetes-associated differences in fibrin content on the examined procedural outcomes of mechanical thrombectomy was identified. These findings suggest that although diabetes is associated with a distinct quantitative change in cerebral thrombus composition, a corresponding procedural consequence was not detectable in the present study.

## 1. Introduction

Diabetes is a systemic metabolic disorder with profound effects on thrombosis and hemostasis [1,2]. Beyond its well-established effects on platelet activation, coagulation, and fibrinolysis [1,2,3], diabetes may also influence the biological and structural characteristics of thrombi that ultimately form within the circulation. Fibrin is of particular interest in this context, as diabetes has been associated with increased fibrinogen levels [1,2], as well as altered fibrin formation and network structure and reduced susceptibility to fibrinolysis [1,3,4,5]. These observations provide a biological basis for hypothesizing that thrombi formed in patients with diabetes may have a distinct composition, particularly with respect to fibrin.

Mechanical thrombectomy provides a unique opportunity to directly examine thrombi responsible for acute ischemic stroke. Previous histological studies have demonstrated substantial heterogeneity in the cellular and molecular composition of retrieved cerebral thrombi, reflecting differences in thrombus origin and the biological conditions under which they form [6,7]. Notably, a previous study reported greater fibrin content in cerebral thrombi retrieved from patients with diabetes, suggesting that the systemic prothrombotic alterations associated with diabetes may indeed be reflected in the composition of occlusive cerebral thrombi [8]. However, direct histological evidence linking diabetes to cerebral thrombus composition remains limited, and the robustness of this association has not been well established.

Beyond its biological relevance, a diabetes-associated change in thrombus composition may also have important procedural implications. Mechanical thrombectomy involves direct physical interaction between thrombectomy devices and the occlusive thrombus, and this interaction is influenced by thrombus properties such as stiffness, deformability, and friction [9]. These properties vary according to thrombus composition, with fibrin-rich thrombi generally exhibiting mechanical characteristics that may render them more resistant to retrieval [6,9,10]. Clinical studies have also suggested an association between diabetes and impaired arterial recanalization following reperfusion therapy. Diabetes has been associated with lower rates of recanalization after intravenous thrombolysis and with unsuccessful reperfusion after mechanical thrombectomy [11,12]. Thus, if diabetes alters cerebral thrombus composition toward greater fibrin content, an important clinical question is whether this compositional change contributes to the procedural outcomes of mechanical thrombectomy.

In this study, we sought not only to determine whether diabetes was independently associated with greater fibrin content in cerebral thrombi, but also to explore the procedural implications of this biological relationship in modern endovascular stroke treatment. Specifically, we investigated whether diabetes-associated differences in fibrin content translated into procedural consequences during mechanical thrombectomy by examining fibrin content as a potential mediator between diabetes and procedural outcomes.

## 2. Materials and Methods

### 2.1. Study Population

We retrospectively reviewed patients with acute ischemic stroke who underwent endovascular treatment at a tertiary stroke center (Severance Hospital, Seoul, Republic of Korea) from 1 January 2017 to 31 October 2021. The inclusion criteria were as follows: (1) acute intracranial artery occlusion and (2) treatment with mechanical thrombectomy. The exclusion criteria were (1) unavailability of thrombus histological analysis and (2) specific occlusion etiologies, including intracranial atherosclerosis-related occlusion, in-stent occlusion, arterial dissection, and moyamoya disease. These etiologies were excluded to reduce heterogeneity arising from distinct pathomechanisms that may influence thrombus composition and because their endovascular treatment characteristics and outcomes differ substantially from those of embolic occlusions.

### 2.2. Mechanical Thrombectomy Procedure

Mechanical thrombectomy was performed (by B.M.K. and D.J.K.) according to standard endovascular practice. A stent retriever (Solitaire, Medtronic, Irvine, CA, USA; Trevo, Stryker Neurovascular, Fremont, CA, USA) was used as the first-line approach in most cases, although contact aspiration thrombectomy (Penumbra, Penumbra Inc., Alameda, CA, USA) was used at the discretion of the treating neurointerventionalist. For anterior circulation occlusions, an 8- or 9-F balloon guide catheter (Cello, Fuji Systems Corporation, Tokyo, Japan; Optimo, Tokai Medical Products, Kasugai, Japan) was routinely used, with proximal flow arrest and manual aspiration during stent retriever retrieval. A distal access catheter was used primarily in cases with substantial vascular tortuosity. Concurrent use of a stent retriever and contact aspiration was generally avoided and reserved for selected cases in which standard approaches were unsuccessful. Decisions regarding additional thrombectomy attempts, conversion to an alternative technique, or termination of the procedure were made by the treating neurointerventionalist according to procedural circumstances.

### 2.3. Thrombus Staining and Analysis

Thrombus specimens obtained during mechanical thrombectomy were fixed in 4% paraformaldehyde, embedded in paraffin, and sectioned for histological analysis. When multiple thrombus fragments were available, the largest fragment was used for sectioning and subsequent histological analysis. The number of sections analyzed was determined by the amount and configuration of the retrieved thrombus material rather than by a fixed number of sections per patient. Consecutive sections were used for the respective immunohistochemical stains. Immunohistochemical staining was performed to identify the major thrombus components, with red blood cells (RBCs), platelets, and fibrin labeled using antibodies against glycophorin A, CD42b, and fibrinogen, respectively. Stained sections were digitized using a whole-slide scanner (Leica Biosystems, Richmond, IL, USA) or a Stereo Investigator Imaging system (MBF Bioscience, Williston, VT, USA) coupled to an Axio Imager D2 microscope (Carl Zeiss, Jena, Germany). Thrombus composition was quantified using Automated Region-of-interest-based Image Analysis (ARIA) software [13]. After delineation of the thrombus area, immunohistochemical signals were separated by color deconvolution, and the positively stained area for each component was measured relative to the total thrombus area. Each component was independently quantified on its respective immunostained section. RBC, platelet, and fibrin contents were expressed as percentages of the corresponding total thrombus area. ARIA has previously been validated against conventional image analysis and demonstrated improved reproducibility across analysts with different levels of experience [13].

### 2.4. Study Variables

Diabetes was defined as either a previously established diagnosis of diabetes with ongoing treatment with oral glucose-lowering agents or insulin at the time of admission, or an HbA1c level ≥ 6.5% measured during hospitalization in patients without a prior history of diabetes [14]. Initial glucose was defined as the blood glucose level measured immediately upon presentation to the emergency department. HbA1c was measured within 3 days of admission, with most measurements obtained as part of routine laboratory testing on the day following admission. Onset-to-puncture time was defined as the interval from symptom onset or last known well to arterial puncture for mechanical thrombectomy. Procedural outcomes included successful reperfusion, complete reperfusion, first-pass effect, and the number of thrombectomy passes. The degree of reperfusion was assessed using the modified Thrombolysis in Cerebral Infarction (mTICI) scale. Successful reperfusion was defined as a final mTICI grade of 2b or 3, whereas complete reperfusion was defined as a final mTICI grade of 3. First-pass effect was defined as achieving mTICI 2c or 3 after a single thrombectomy pass, sustained without additional intervention [15].

### 2.5. Statistical Analysis

Statistical analyses were conducted in a stepwise manner. First, clinical characteristics and thrombus composition were compared between patients with and without diabetes to characterize the study groups and assess differences in thrombus composition according to diabetes status. Continuous variables were compared using Student’s *t*-test or Mann–Whitney *U* test, as appropriate, and categorical variables using the Pearson chi-square test or Fisher exact test. Subsequent analyses focused on characterizing the association between diabetes and fibrin content.

Second, the association between diabetes and fibrin content was evaluated using linear regression, with fibrin content as the dependent variable and diabetes status as the primary independent variable. Regression coefficients for diabetes were expressed in percentage points (%*p*), representing the estimated difference in fibrin content associated with diabetes. Covariates for multivariable adjustment were prespecified based on clinical relevance and their potential associations with diabetes or thrombus composition, rather than selected according to statistical significance in univariable analyses. Model 1 was adjusted for age, sex, atrial fibrillation, and intravenous tPA administration. Age and sex were included as demographic covariates. Atrial fibrillation was included because it is an important clinical factor associated with differences in thrombus composition. Intravenous tPA administration was included because preprocedural thrombolytic exposure may alter the composition of thrombi subsequently analyzed histologically. Model 2 additionally included initial glucose as a complementary analysis to examine whether the diabetes–fibrin association persisted after conditioning on glycemic status at presentation. Initial glucose was not considered a conventional confounder, as it may partly reflect the metabolic consequences of diabetes and potentially lie on a pathway linking diabetes to thrombus characteristics. Rather, this additional model was intended to distinguish the association with diabetes as a chronic disease state from that observed after accounting for glycemic status at presentation. For linear regression models, model assumptions were assessed using residual diagnostics and evaluation of influential observations.

Third, sensitivity analyses were performed to assess the robustness of the association between diabetes and fibrin content with respect to thrombolytic exposure and atrial fibrillation status. In the first sensitivity analysis, the cohort was restricted to patients who did not receive intravenous tPA, thereby excluding the potential influence of preprocedural thrombolysis on the histologically analyzed thrombus. The model was adjusted for age, sex, atrial fibrillation, and initial glucose. In the second sensitivity analysis, the cohort was restricted to patients with atrial fibrillation to assess whether the diabetes–fibrin association remained consistent within this clinically defined subgroup. Because atrial fibrillation was constant within this cohort, the model was adjusted for age, sex, intravenous tPA administration, and initial glucose. To further characterize the observed diabetes–fibrin association, the relationships of HbA1c and initial glucose with fibrin content were additionally examined among patients with diabetes using separate univariable linear regression models.

Fourth, formal mediation analyses were performed to estimate the indirect effects of diabetes on procedural outcomes through diabetes-associated changes in fibrin content. Diabetes was specified as the exposure, fibrin content as the mediator, and each procedural measure as the outcome. The outcomes examined were successful reperfusion, complete reperfusion, first-pass effect, number of thrombectomy passes, and ≥3 thrombectomy passes. These interrelated outcomes were examined as complementary measures of thrombectomy performance, without a prespecified primary procedural endpoint. Given the exploratory nature of the mediation analyses, no adjustment for multiple comparisons was applied. The mediator model estimated the association between diabetes and fibrin content, whereas each outcome model estimated the associations of diabetes and fibrin content with the respective procedural outcome. All models were adjusted for age, sex, atrial fibrillation, intravenous tPA administration, and initial glucose. The mediator model was fitted using linear regression. For the outcome models, logistic regression was used for binary outcomes and linear regression for the number of passes. Together, the mediator and outcome models were used to estimate the indirect association between diabetes and each procedural outcome through fibrin content. The natural indirect effect (NIE) was then estimated to quantify this mediated pathway [16]. Thus, the NIE characterizes the estimated indirect pathway from diabetes through fibrin content to the procedural outcome, rather than the overall effect of diabetes on the outcome or the association between fibrin content and the outcome alone. Causal interpretation of the estimated indirect effects requires adequate control of exposure–outcome, exposure–mediator, and mediator–outcome confounding, as well as the absence of mediator–outcome confounders affected by the exposure. Given the retrospective observational design, these assumptions cannot be fully verified. Accordingly, the estimated indirect effects were interpreted primarily as characterizing an associational mediated pathway through fibrin content rather than establishing a causal mediation effect. For binary outcomes, NIEs were reported as odds ratios (ORs); for the number of passes, the NIE was reported as a regression coefficient (β). The 95% confidence intervals (CIs) and *p* values were estimated using 5000 nonparametric bootstrap resamples.

All statistical analyses were performed using R software (version 4.5.1; R Foundation for Statistical Computing, Vienna, Austria). A two-sided *p* value < 0.05 was considered statistically significant.

## 3. Results

**Figure 1:**
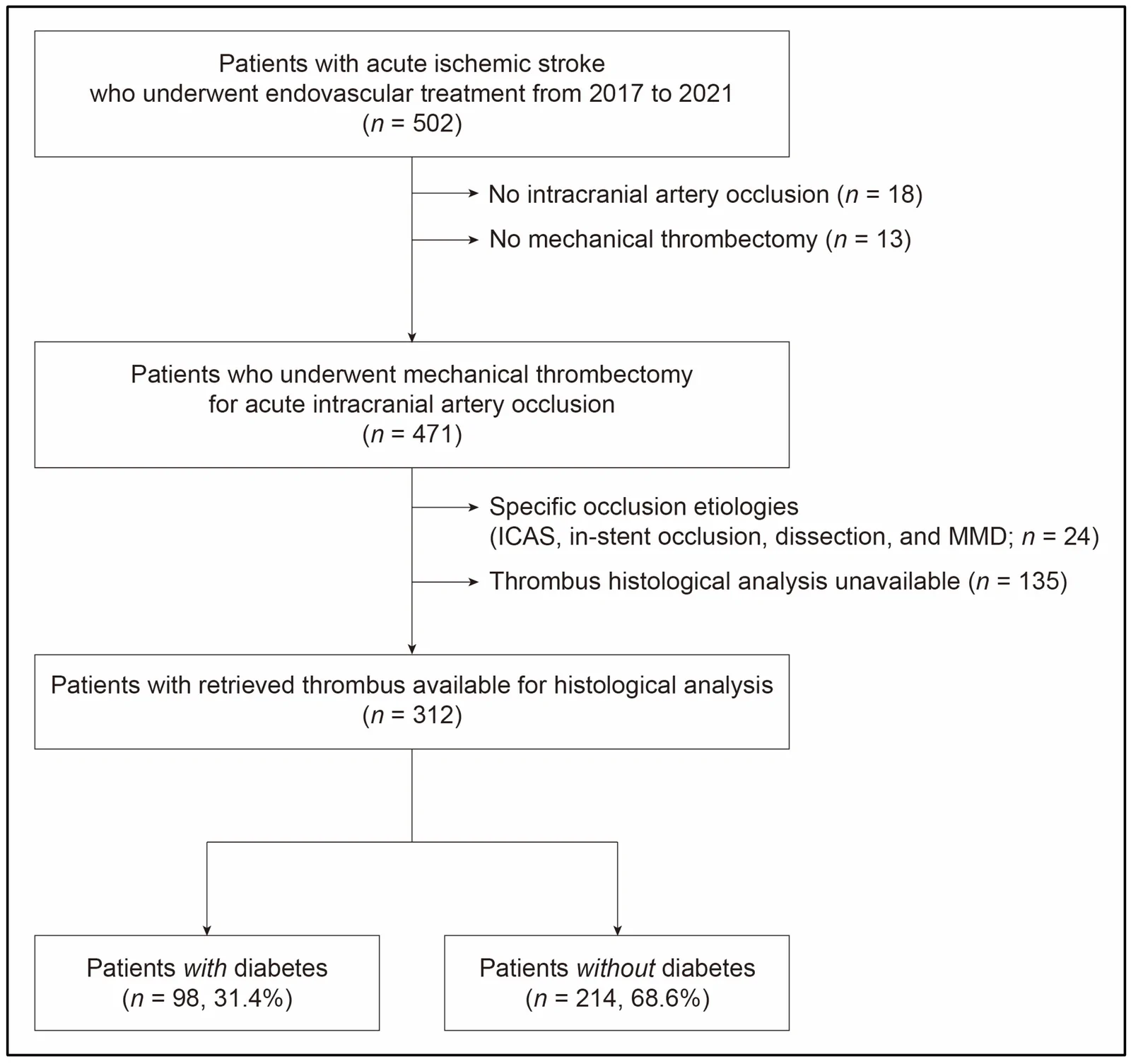
Flowchart of study population selection. ICAS, intracranial atherosclerosis; MMD, moyamoya disease.

Among 502. patients with acute ischemic stroke who underwent endovascular treatment during the study period, 190 were excluded during the study population selection process, leaving 312 patients for the final analysis (Figure 1). Of these, 98 (31.4%) had diabetes and 214 (68.6%) did not. Clinical and laboratory characteristics were compared according to diabetes status (Table 1). Fibrin content was higher in patients with diabetes than in those without diabetes (median, 37.0% [interquartile range, 26.4–52.9] vs. 30.9% [17.7–45.0]; *p* = 0.004), whereas RBC and platelet contents did not differ significantly between the groups.

### 3.1. Independent Association Between Diabetes and Fibrin Content

To evaluate whether the difference in fibrin content according to diabetes status persisted after accounting for relevant clinical factors, linear regression analyses were performed (Table 2). In the unadjusted model, diabetes was associated with a 6.58%*p* higher fibrin content (95% CI, 2.22–10.93; *p* = 0.003). In the primary adjusted model (Model 1), diabetes remained associated with a 6.68%*p* higher fibrin content (95% CI, 2.19–11.18; *p* = 0.004). The association remained evident in Model 2, which additionally conditioned on initial glucose (+8.32%*p*; 95% CI, 3.27–13.37; *p* = 0.001).

**Figure 2:**
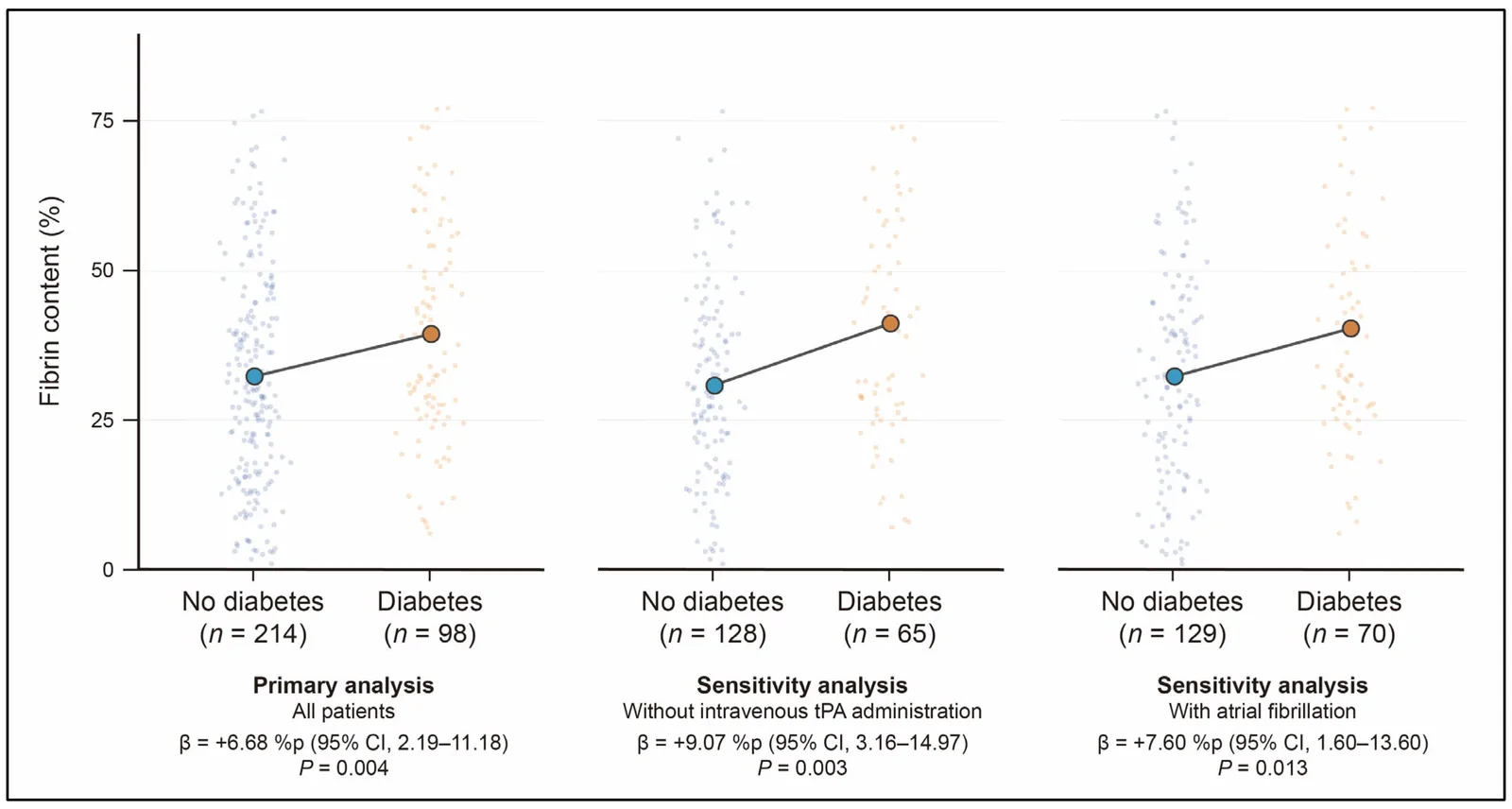
Adjusted fibrin content according to diabetes status in the primary and sensitivity analyses. Small dots represent individual fibrin content values, and large circles represent adjusted mean fibrin content estimated from linear regression models. The primary analysis included the entire study cohort and was adjusted for age, sex, atrial fibrillation, and intravenous tPA administration. The sensitivity analysis restricted to patients without intravenous tPA administration was adjusted for age, sex, atrial fibrillation, and initial glucose. The sensitivity analysis restricted to patients with atrial fibrillation was adjusted for age, sex, intravenous tPA administration, and initial glucose. β coefficients represent the adjusted difference in fibrin content associated with diabetes. CI, confidence interval; tPA, tissue plasminogen activator.

Sensitivity analyses were performed to assess whether the association remained consistent after restricting the analysis based on thrombolytic exposure and atrial fibrillation status (Table 3). Among patients who did not receive intravenous tPA, diabetes was associated with a 9.07%*p* higher fibrin content after adjustment (95% CI, 3.16–14.97; *p* = 0.003). Similarly, among patients with atrial fibrillation, diabetes was associated with a 7.60%*p* higher fibrin content after adjustment (95% CI, 1.60–13.60; *p* = 0.013). Adjusted fibrin content was consistently higher in patients with diabetes across the primary and sensitivity analyses (Figure 2).

Among patients with diabetes, neither HbA1c (β = −0.13%*p* per 1% increase; 95% CI, −2.62 to 2.35; *p* = 0.915) nor initial glucose (β = −0.023%*p* per 1 mg/dL increase; 95% CI, −0.079 to 0.033; *p* = 0.414) was significantly associated with fibrin content.

### 3.2. Indirect Effects of Diabetes on Procedural Outcomes Through Fibrin Content

NIEs of diabetes through fibrin content were estimated for five procedural outcomes (Table 4). For reperfusion outcomes, the NIEs—representing the effects of diabetes transmitted through diabetes-associated changes in fibrin content—were estimated as OR 0.98 (95% CI, 0.79–1.25; *p* = 0.898) for successful reperfusion, OR 1.00 (95% CI, 0.88–1.12; *p* = 0.959) for complete reperfusion, and OR 1.01 (95% CI, 0.90–1.14; *p* = 0.803) for first-pass effect. For the number of passes, the NIE was β = −0.050 (95% CI, −0.161 to 0.038; *p* = 0.282); for ≥3 passes, the corresponding OR was 0.96 (95% CI, 0.83–1.08; *p* = 0.474). None of the indirect effects reached statistical significance, with point estimates close to the null.

## 4. Discussion

In this study, diabetes was associated with greater fibrin content in cerebral thrombi retrieved during mechanical thrombectomy after adjustment for relevant clinical factors. This association persisted after additional conditioning on glucose at presentation and in sensitivity analyses restricted to patients without intravenous tPA administration and to those with atrial fibrillation. In contrast, neither HbA1c nor initial glucose was associated with fibrin content among patients with diabetes. Despite the robust association between diabetes and fibrin content, mediation analyses did not provide evidence of a detectable indirect effect through fibrin content on the examined procedural outcomes. Taken together, our findings support the presence of a fibrin-enriched cerebral thrombus phenotype associated with diabetes, but no detectable fibrin-mediated effect on thrombectomy procedural outcomes was identified.

There is a strong biological basis for the association between diabetes and fibrin content observed in our study. Diabetes affects multiple aspects of fibrin biology through a complex prothrombotic milieu involving increased fibrinogen availability, altered fibrin formation and polymerization, changes in fibrin network architecture, and impaired fibrinolysis [1,2,3,4,5]. These abnormalities can promote the formation of denser and less permeable fibrin networks with greater resistance to fibrinolysis [1,3,4,5]. However, this body of evidence has predominantly characterized structural, functional, and fibrinolytic properties of fibrin rather than the quantitative contribution of fibrin to an established thrombus. Moreover, much of the evidence has been derived from circulating biomarkers, plasma-based assays, and experimental models rather than from thrombi formed in vivo under clinical conditions [1,3,5]. Our findings extend this biological framework into both quantitative and clinical domains by demonstrating that diabetes is associated with greater fibrin content in cerebral thrombi retrieved from patients with acute ischemic stroke.

Direct quantitative histological evidence from cerebral thrombi, however, remains limited. A previous histological study reported greater fibrin content in thrombectomy-retrieved cerebral thrombi from patients with diabetes [8]. A subsequent study focusing on diabetes accompanied by admission hyperglycemia similarly demonstrated a fibrin-enriched composition, along with differences in thrombus ultrastructure and perviousness [17]. Although these findings support a diabetes-associated cerebral thrombus phenotype, the available evidence has been derived from relatively small cohorts. The consistent association across multivariable and sensitivity analyses accounting for relevant clinical factors and examining acute glycemic status, thrombolytic exposure, and atrial fibrillation status further supports a robust relationship between diabetes and greater fibrin content in cerebral thrombi.

Of note, previous studies have linked hyperglycemia and poor glycemic control to altered fibrin properties, and a fibrin-enriched cerebral thrombus pattern has been reported in patients with diabetes accompanied by admission hyperglycemia [1,3,17]. In our study, however, neither initial glucose nor HbA1c was associated with fibrin content among patients with diabetes, while the association between diabetes and fibrin content persisted after conditioning on initial glucose. These null associations should be interpreted cautiously given the modest sample size of the diabetes subgroup and the resulting potential for sampling variability and limited statistical power. Moreover, a single glucose measurement at admission may not adequately reflect chronic glycemic exposure or the broader metabolic state relevant to thrombus formation. Fibrin content in an established thrombus may instead reflect the cumulative influence of multiple metabolic and prothrombotic processes associated with diabetes. Indeed, longer diabetes duration has been associated with a more prothrombotic fibrin phenotype independently of glycemic control [18].

Experimental and clinical evidence suggests that fibrin-rich thrombi may exhibit unfavorable mechanical properties, including greater stiffness and friction, making them more difficult to retrieve [6,9,10]. However, the fibrin-enriched histological phenotype observed in our study should be distinguished from thrombus mechanical behavior, which was not directly measured. Conceptually, diabetes-associated differences in thrombus composition may influence procedural outcomes through changes in mechanical properties such as stiffness, friction, and deformability, providing a potential pathway linking diabetes to thrombectomy performance. Because these intermediate mechanical properties were not assessed in the present study, this mechanistic pathway remains hypothesis-generating and requires direct evaluation in future studies. In the present study, we examined whether diabetes-associated differences in fibrin content were associated with procedural outcomes through mediation analysis. Despite the robust diabetes–fibrin association, our mediation analyses did not provide evidence of a detectable fibrin-mediated effect on any of the examined procedural outcomes. Several factors may explain this apparent disconnect. First, the magnitude of the diabetes-associated increase in fibrin may be sufficient to define a distinct histological phenotype but insufficient to produce a measurable change in procedural performance. Second, fibrin content represents only one quantitative dimension of thrombus composition and does not capture fibrin architecture, organization, or its interactions with other thrombus components, all of which may substantially influence mechanical behavior [6,9]. Thrombus components are inherently interrelated within a finite thrombus structure, and a difference in the relative abundance of one component may therefore be accompanied by changes in others. Accordingly, the absence of statistically significant differences in RBC or platelet content should not be interpreted as evidence that the observed difference in fibrin content occurred independently of other compositional changes. Finally, procedural outcomes reflect not only thrombus properties but also vascular anatomy, device–thrombus interaction, thrombectomy technique, and other procedural factors, which may dilute the measurable contribution of diabetes-associated differences in fibrin content. Thus, the absence of a detectable indirect effect should not be interpreted as evidence that fibrin is irrelevant to thrombectomy mechanics; rather, it suggests that the quantitative difference in fibrin associated with diabetes may not be sufficient to mediate a measurable effect of diabetes on procedural outcomes. However, the confidence intervals around several indirect-effect estimates remained compatible with modest effects, and the present findings therefore do not exclude a smaller fibrin-mediated procedural effect.

A broader implication of these findings is that disease-associated changes in thrombus composition and their procedural consequences should be considered related but distinct phenomena. The presence of a measurable histological signature of diabetes does not necessarily imply a corresponding difference in thrombectomy performance. By demonstrating a robust association between diabetes and greater fibrin content without a detectable fibrin-mediated effect on procedural outcomes, our study suggests that the clinical relevance of a disease-associated thrombus phenotype may not be fully captured by quantitative composition alone. Future studies integrating quantitative composition with structural and mechanical characteristics may help determine how diabetes-associated thrombus phenotypes translate into clinically relevant behavior during endovascular treatment.

Several limitations should be acknowledged. First, this was a retrospective, single-center study, and residual confounding cannot be excluded despite multivariable adjustment and sensitivity analyses. Second, thrombus histological analysis was unavailable in a substantial proportion of otherwise eligible patients, which may have introduced selection bias. The reasons for the unavailability of histological analysis could not be fully assessed. Therefore, we could not determine whether histological availability was associated with thrombus retrieval, fragmentation, procedural circumstances, or other patient- or treatment-related factors. Accordingly, the analyzed cohort may not fully represent the overall population of patients undergoing mechanical thrombectomy. Third, because patients with intracranial atherosclerosis-related occlusion, in-stent occlusion, dissection, and moyamoya disease were excluded, the present findings should be interpreted primarily within the selected thrombectomy population rather than generalized to cerebral thrombi across all ischemic stroke etiologies. Fourth, formal stroke-etiology classification was not available for reliable use in the present analysis. Although atrial fibrillation was considered because of its relevance to thrombus composition, it is not synonymous with cardioembolic stroke and does not fully account for etiologic heterogeneity within the study population. Fifth, our histological analysis quantified the relative content of major thrombus components but did not fully capture intrathrombus spatial heterogeneity or assess fibrin architecture, organization, or mechanical properties. This distinction is particularly relevant to the mediation analyses, as fibrin content alone may not capture the thrombus characteristics that determine its interaction with thrombectomy devices. Sixth, the mediation analyses examined several interrelated procedural outcomes without adjustment for multiple comparisons and should therefore be regarded as exploratory. Finally, mediation analysis in an observational study relies on assumptions regarding unmeasured confounding of the exposure–mediator and mediator–outcome relationships. Accordingly, the absence of a detectable indirect effect indicates a lack of evidence for the specific diabetes–fibrin content–procedural outcome pathway examined here, rather than definitive evidence against such a pathway.

## 5. Conclusions

Diabetes was independently associated with greater fibrin content in cerebral thrombi retrieved during mechanical thrombectomy, supporting the presence of a fibrin-enriched thrombus phenotype associated with diabetes. However, no detectable indirect effect of diabetes-associated differences in fibrin content on the examined procedural outcomes of mechanical thrombectomy was identified. These findings suggest that although diabetes is associated with a distinct quantitative change in cerebral thrombus composition, a corresponding procedural consequence was not detectable in the present study.

## Figures and Tables

**Table 1:** Clinical and thrombus characteristics according to diabetes status.

Variables	No Diabetes (*n* = 214)	Diabetes (*n* = 98)	*p* Value
**Clinical characteristics**			
Age, years	69.6 ± 13.5	74.7 ± 8.9	<0.001
Male	99 (46.3)	51 (52.0)	0.343
Hypertension	136 (63.6)	87 (88.8)	<0.001
Dyslipidemia	58 (27.1)	38 (38.8)	0.038
Current smoking	26 (12.1)	15 (15.3)	0.444
Atrial fibrillation	129 (60.3)	70 (71.4)	0.057
Initial NIHSS score	13 [9–18]	16 [12–20]	<0.001
Intravenous tPA administration	86 (40.2)	33 (33.7)	0.272
Occlusion site			0.683
Internal carotid artery	58 (27.1)	29 (29.6)	
Middle cerebral artery			
M1 segment	82 (38.3)	32 (32.7)	
M2 segment	53 (24.8)	23 (23.5)	
Basilar artery	16 (7.5)	10 (10.2)	
Others	5 (2.3)	4 (4.1)	
Onset-to-puncture time, minutes	255 [135–545]	244 [150–564]	0.613
**Diabetes-related laboratory findings**			
Initial glucose, mg/dL	121 [106–140]	158 [130–197]	<0.001
HbA1c, %	5.7 [5.4–6.0]	6.5 [6.0–7.6]	<0.001
**Thrombus composition**			
Red blood cell, %	37.1 [19.0–47.9]	35.4 [24.1–43.7]	0.914
Platelet, %	8.9 [3.6–16.7]	9.3 [4.4–19.9]	0.308
Fibrin, %	30.9 [17.7–45.0]	37.0 [26.4–52.9]	0.004

Values are presented as mean ± standard deviation, median [interquartile range], or *n* (%), as appropriate. HbA1c, glycated hemoglobin; NIHSS, National Institutes of Health Stroke Scale; tPA, tissue plasminogen activator.

**Table 2:** Linear regression analysis of the association between diabetes and fibrin content.

Model	β for Diabetes, %*p* (95% CI)	*p* Value
Unadjusted model	+6.58 (2.22–10.93)	0.003
Adjusted models		
Model 1	+6.68 (2.19–11.18)	0.004
Model 2	+8.32 (3.27–13.37)	0.001

β coefficients were estimated using linear regression and represent the difference in fibrin content (percentage points, %*p*) associated with diabetes. Model 1 was the primary adjusted model and included age, sex, atrial fibrillation, and intravenous tPA administration. Model 2 additionally included initial glucose to examine the association after conditioning on glycemic status at presentation. CI, confidence interval; tPA, tissue plasminogen activator.

**Table 3:** Sensitivity analyses of the association between diabetes and fibrin content using linear regression.

Subgroup	β for Diabetes, %*p* (95% CI)	*p* Value
Without intravenous tPA administration	+9.07 (3.16–14.97)	0.003
With atrial fibrillation	+7.60 (1.60–13.60)	0.013

β coefficients were estimated using linear regression and represent the difference in fibrin content (percentage points, %*p*) associated with diabetes. Analyses were adjusted for age, sex, atrial fibrillation, and initial glucose in patients without intravenous tPA administration, and for age, sex, intravenous tPA administration, and initial glucose in patients with atrial fibrillation. CI, confidence interval; tPA, tissue plasminogen activator.

**Table 4:** Mediation analysis of diabetes and procedural outcomes with fibrin content as the mediator.

Procedural Outcome	Natural Indirect Effect Through Fibrin Content (95% CI)	*p* Value
Successful reperfusion	OR 0.98 (0.79–1.25)	0.898
Complete reperfusion	OR 1.00 (0.88–1.12)	0.959
First-pass effect	OR 1.01 (0.90–1.14)	0.803
Number of passes	β −0.050 (−0.161 to 0.038)	0.282
≥3 passes	OR 0.96 (0.83–1.08)	0.474

Natural indirect effects represent the effects of diabetes on procedural outcomes mediated through diabetes-associated changes in fibrin content. The mediator and outcome models were adjusted for age, sex, atrial fibrillation, intravenous tPA administration, and initial glucose. Binary outcomes were modeled using logistic regression, whereas number of passes was modeled using linear regression. The 95% CIs and *p* values were estimated using 5000 nonparametric bootstrap resamples. Effect estimates are presented as ORs for binary outcomes and as a regression coefficient (β) for number of passes. Successful reperfusion was defined as final mTICI grade 2b or 3, and complete reperfusion as final mTICI grade 3. CI, confidence interval; mTICI, modified Thrombolysis in Cerebral Infarction; OR, odds ratio.

## Data Availability

The data presented in this study are available on request from the corresponding author.
